# Canard solutions in neural mass models: consequences on critical regimes

**DOI:** 10.1186/s13408-021-00109-z

**Published:** 2021-09-16

**Authors:** Elif Köksal Ersöz, Fabrice Wendling

**Affiliations:** Univ Rennes, INSERM, LTSI-U1099, Campus de Beaulieu, F - 35000 Rennes, France

**Keywords:** 34E15, 34E17, 37N25, 92B25, Multiple time-scale systems, Canards, Bursting, Excitability, Epilepsy, Neural mass model

## Abstract

Mathematical models at multiple temporal and spatial scales can unveil the fundamental mechanisms of critical transitions in brain activities. Neural mass models (NMMs) consider the average temporal dynamics of interconnected neuronal subpopulations without explicitly representing the underlying cellular activity. The mesoscopic level offered by the neural mass formulation has been used to model electroencephalographic (EEG) recordings and to investigate various cerebral mechanisms, such as the generation of physiological and pathological brain activities. In this work, we consider a NMM widely accepted in the context of epilepsy, which includes four interacting neuronal subpopulations with different synaptic kinetics. Due to the resulting three-time-scale structure, the model yields complex oscillations of relaxation and bursting types. By applying the principles of geometric singular perturbation theory, we unveil the existence of the canard solutions and detail how they organize the complex oscillations and excitability properties of the model. In particular, we show that boundaries between pathological epileptic discharges and physiological background activity are determined by the canard solutions. Finally we report the existence of canard-mediated small-amplitude frequency-specific oscillations in simulated local field potentials for decreased inhibition conditions. Interestingly, such oscillations are actually observed in intracerebral EEG signals recorded in epileptic patients during pre-ictal periods, close to seizure onsets.

## Introduction

Brain dynamics emerges from neural entities interacting at different levels, from single neurons to large-scale neural networks. At each level, transitions between different regimes, such as firing/resting states in single neurons and up/down states in neural networks, are associated with both physiological functions and pathological activity [1–3]. One of the features of the system that determines how these transitions would occur is *excitability*. The concept of neural excitability for single neurons was introduced initially by Louis Lapique in 1907 [4, 5]. Alan Hodgkin, who then *re-introduced* the concept [6], classified the neural excitability with respect the firing rate of neurons in response to injected steps of currents. Excitability properties of neural systems can vary with internal dynamics, leading to different physiological and pathological behavior [7–10]. At the cortical scale, for instance, variations in excitability [11] and loss of network resilience [12] are associated with epileptic seizures. Yet, what may be as important as a transition itself is the dynamics preceding the transition. In the context of epilepsy, for example, identification of the dynamic features along the path to a transition is crucial for intervention and prevention of seizures.

Mathematical models of brain activity range from microscopic level of single cell dynamics to macroscopic level of interactions between large scale neural systems. Neural mass models (NMMs) consider the average temporal dynamics of interconnected neural subpopulations without explicitly representing the underlying mechanisms at the level of single cells. The mesoscopic level offered by the neural mass formulation has been used to model brain signals, from local field potentials (LFPs) to global electroencephalographic (EEG) recordings, and to investigate various cerebral rhythms [13–15]. NMMs have also been used extensively to study pathological dynamics such as in epilepsy [16–19], Alzheimer’s disease [20] and Parkinson’s disease [21, 22].

Interactions between slow and fast components of neural systems, hence, of their mathematical models, result in multiple time-scale complex oscillations, such as relaxation, bursting and mixed-mode oscillations. Geometric singular perturbation theory (GSPT) is a key tool for understanding the interaction between the geometry of the system and the emerging multiple time-scale dynamics. In particular, canard solutions, which can exist in multiple time-scale systems with a folded geometry, appear as building blocks of complex oscillations in both phenomenological and neurophysiologically plausible models ranging from single cell [23–26] to neural networks [27, 28]. The canard phenomenon in such systems has been related to neural excitability [29], excitability thresholds [23, 30–34], and boundaries between different type of solutions, such as subthreshold oscillations and large amplitude spiking/bursting oscillations [24, 28, 35–43]. While such canard-organized fine structures have been shown in a wide range of two-time-scale models, recent studies started to explore canard-mediated processes in systems with three or more time-scales [44–46].

**Figure 1:**
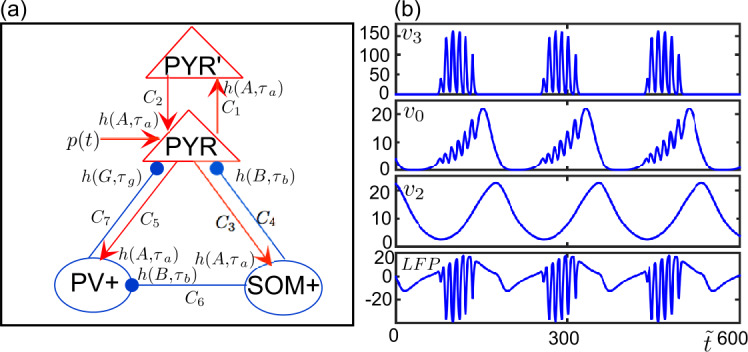
Model diagram and time series of a bursting solution. (**a**) Model diagram showing excitatory (red connections) and inhibitory (blue connections) interactions between subpopulations of pyramidal neurons (PYR and PYR’) and inhibitory interneurons (PV+ and SOM+). The post-synaptic potential of a subpopulation, which is the output of $h(t)$, is multiplied by a synaptic coefficient $C_{i}$ before being received by another subpopulation. (**b**) Time series of a bursting solution for the parameter set in Table 1. The panels from the top to the bottom show the time courses of post-synaptic potentials of PV+ ($v_{3}$), PYR ($v_{0}$), SOM+ ($v_{2}$) and the local field potential (LFP), i.e. $A \tau _{a} p + C_{2} \tau _{a} v_{1}- C_{4} \tau _{b} v_{2} - C_{7} \tau _{g} v_{3}$

In this study we investigate critical regimes in the NMM initially presented in [16]. This physiologically-grounded model has been extensively used for modeling structural and functional changes leading to epileptic activity observed in intracranial (stereoelectroencephalography, SEEG) signals. The model includes four interacting neuronal subpopulations: two interconnected subpopulations of glutamatergic pyramidal neurons and GABAergic inhibitory interneurons (somatostatin positive (SOM+), and parparvalbunim positive (PV+), also called dendrite-projecting slow and soma-projecting fast interneurons, respectively). Although the model was introduced for the CA1 region of the hippocampus, implementation of these four subpopulations mediating glutamatergic and GABAergic signaling makes it generic enough to be considered for many other cortical regions [47]. Activity of each subpopulation is given by the corresponding average post-synaptic potential (PSP) that is determined by two functions: 1) a “pulse to wave” function, $S(v)=5/(1+\exp (0.56(6-v)))$, transforming the incoming synaptic potentials into a firing rate; and 2) a “wave to pulse” converting the input average firing rate into a mean PSP at the input of each subpopulation, that is, $h(t)=W t /\tau _{w} \exp (-t/\tau _{w})$, where *W* represents the average synaptic gain and $\tau _{w}$ is the average synaptic time constant mimicking the rise and decay of actual PSPs. The system schematized in Fig. 1a reads

1a $$\begin{aligned}& \dot{y}_{0} = y_{5}, \end{aligned}$$

1b $$\begin{aligned}& \dot{y}_{5} = \frac{A}{\tau _{a}}S[y_{1} - y_{2} - y_{3}] - \frac{2}{\tau _{a}} y_{5} - \frac{1}{\tau _{a}^{2}}y_{0}, \end{aligned}$$

1c $$\begin{aligned}& \dot{y}_{1} = y_{6} \end{aligned}$$

1d $$\begin{aligned}& \dot{y}_{6} = \frac{A}{\tau _{a}}\bigl\{ p(t) + C_{2} S[C_{1}y_{1}]\bigr\} - \frac{2}{\tau _{a}}{y}_{6} - \frac{1}{\tau _{a}^{2}}y_{1}, \end{aligned}$$

1e $$\begin{aligned}& \dot{y}_{2} = y_{7}, \end{aligned}$$

1f $$\begin{aligned}& \dot{y}_{7} = \frac{B}{\tau _{b}} C_{4} S[C_{3}y_{0}] - \frac{2}{\tau _{b}}y_{7} - \frac{1}{\tau _{b}^{2}}y_{2}, \end{aligned}$$

1g $$\begin{aligned}& \dot{y}_{3} = y_{8}, \end{aligned}$$

1h $$\begin{aligned}& \dot{y}_{8} = \frac{G}{\tau _{g}} C_{7} S \biggl[C_{5}y_{0} - \frac{C_{6}}{C_{4}}y_{4}\biggr] - \frac{2}{\tau _{g}}y_{8} - \frac{1}{\tau _{a}^{2}}y_{3}. \end{aligned}$$

The variables $y_{0,1}$ stand for the excitatory PSPs mediated by two pyramidal neuron subpopulations, $y_{2}$ and $y_{3}$ are inhibitory PSPs mediated by the SOM+ and PV+ interneuron subpopulations, respectively. Variables $y_{j}$ ($j\in \{ 5,6,7,8\}$) are the auxiliary variables that are introduced to convert the second-order differential equations describing the wave to pulse functions to first-order differential equations [13]. The parameters *A*, *B*, *G* are the synaptic gains, the $C_{i}$ are the connectivity constants representing the average number of synaptic contacts, $p(t)$ is the external (noisy) cortical input ($p(t)= p+\xi $, where *p* is the mean of external input *ξ* is a random variable following a normal distribution with $\mathcal{N}(0,\sigma ^{2})$). The synaptic time constants are given by $\tau _{a}$, $\tau _{b}$, $\tau _{g}$. The major contribution to LFPs (as recorded by intracranial electrodes in patients candidate to surgery) corresponds to the PSPs summated at the level of pyramidal neurons, which are geometrically aligned “in palisades”, i.e. one relative to the other and perpendicular to the plane of the cortical layers. In the model, the LFP is given by the sum of excitatory PSP (EPSP) and inhibitory PSPs (IPSPs) received by the glutamatergic pyramidal cells, hence $\text{LFP} = y_{1} - y_{2} - y_{3}$.

As introduced in [48], under the following variable conversion:

$$ \biggl(\frac{t}{\tau _{g}}, \frac{y_{0}}{\tau _{a}}, \frac{y_{1}}{\tau _{a}}, \frac{y_{2}}{\tau _{b}}, \frac{y_{3}}{\tau _{g}}, y_{5}, y_{6}, y_{7}, y_{8} \biggr) \implies (\tilde{t}, v_{0}, v_{1}, v_{2}, v_{3}, y_{5}, y_{6}, y_{7}, y_{8}), $$

with $\delta = \tau _{g}/\tau _{a} $ and $\varepsilon = \tau _{a}/\tau _{b}$, system (1a)–(1h) can be written in the following deterministic ($\sigma = 0$) slow–fast form:

2a $$\begin{aligned}& \frac{d v_{3}}{d\tilde{t}} = y_{8} := F_{3}(y_{8}), \end{aligned}$$

2b $$\begin{aligned}& \frac{d y_{8}}{d\tilde{t}} = G S[C_{5} \tau _{a} v_{0} - C_{6} \tau _{b} v_{2} ] - v_{3} - 2 y_{8} := F_{8}(v_{0}, v_{2}, v_{3}, y_{8}), \end{aligned}$$

2c $$\begin{aligned}& \frac{d v_{0}}{d\tilde{t}} = \delta y_{5} := \delta F_{0}(y_{5}), \end{aligned}$$

2d $$\begin{aligned}& \begin{aligned}[t] \frac{d y_{5}}{d\tilde{t}} & = \delta \bigl(A S[A \tau _{a} p + C_{2} \tau _{a} v_{1}- C_{4} \tau _{b} v_{2} - C_{7} \tau _{g} v_{3}]-v_{0} - 2 y_{5}\bigr) \\ & := \delta F_{5}(v_{0}, v_{1}, v_{2}, v_{3}, y_{5}), \end{aligned} \end{aligned}$$

2e $$\begin{aligned}& \frac{d v_{1}}{d\tilde{t}} = \delta y_{6} := \delta F_{1}(y_{6}), \end{aligned}$$

2f $$\begin{aligned}& \frac{d y_{6}}{d\tilde{t}} = \delta \bigl(A S[C_{1} \tau _{a} v_{0}] - v_{1} - 2y_{6}\bigr) := \delta F_{6}(v_{0}, v_{1}, y_{6}), \end{aligned}$$

2g $$\begin{aligned}& \frac{d v_{2}}{d\tilde{t}} = \delta \varepsilon y_{7} := \delta \varepsilon F_{2}(y_{7}), \end{aligned}$$

2h $$\begin{aligned}& \frac{d y_{7}}{d\tilde{t}} = \delta \varepsilon \bigl(B S[C_{3} \tau _{a} v_{0}] - v_{2} - 2 y_{7}\bigr) := \delta \varepsilon F_{7}(v_{0}, v_{2}, y_{7}). \end{aligned}$$

In this manuscript, we will consider system (2a)–(2h) for the slow–fast analysis and (1a)–(1h) for simulations under the stochastic input. We will be using the parameter set given in Table 1, unless otherwise stated, for which $\delta = 0.3$ and $\varepsilon = 0.2$. Numerical bifurcation analysis is performed in AUTO-07p software [49]. The stochastic differential equations were integrated using the Euler–Murayama method with a step size $dt = 10\text{e}{-}4$ second in XPPAUT software [50].

**Table 1:** Parameter values for the bursting-type discharges

*A* (mV)	*B* (mV)	*G* (mV)	*p* (Hz)	$C_{1}$	$C_{2}$	$C_{3}$	$C_{4}$	$C_{5}$	$C_{6}$	$C_{7}$	$\tau _{a}$ (s)	$\tau _{b}$ (s)	$\tau _{g}$ (s)
5	5	35	90	135	108	80	25	450	121	121	0.01	0.05	0.003

As noticed in [48], system (2a)–(2h) is a three-time-scale system written in *fast form* with $(v_{3}, y_{8})$ being fast, $(v_{0}, y_{5}, v_{1}, y_{6})$ slow and $(v_{2}, y_{7})$ super-slow variables. Köksal Ersöz et al. [48] have focused on electrophysiological pre-ictal bursting patterns recorded in human patients just before the onset of seizure. Pre-ictal bursting patterns are characterized by fast oscillatory discharges (which will be referred as *spikes*) followed by a slower oscillation (a simulated pattern with the parameter set in Table 1 is exemplified in Fig. 1b). The authors have reproduced pre-ictal bursting and unveiled the mechanism yielding these solutions by decorticating the three-time-scale structure of the model. They have discussed appropriate stimulation strategies for aborting of the pre-ictal bursting, hence, for preventing a subsequent epileptic seizure. However, they did not focus on possible slow–fast transitions. Here we extend the slow–fast analysis initiated in [48] by investigating the role of slow-manifolds in transitions to relaxation and bursting type of solutions. We will focus on how canard trajectories shape the different routes from physiological to pathological brain activity. In what follows, we will go briefly through the multiple-time-scale analysis presented in [48], and then show different canard structures present in the model and how they take a part in critical transitions. Finally, we will see the system’s response to stochastic inputs near critical regimes, and make a remark on the slow oscillations observed along the path to seizure in SEEG signals recorded during pre-surgical evaluation of two patients with drug-resistant epilepsy.

## Preliminaries

System (2a)–(2h) expressed in the fast time *t̃* is called a *fast system*. The *slow system* is obtained by defining $\tilde{t}_{s} = \delta \tilde{t}$,

3a $$\begin{aligned}& \delta \frac{d v_{3}}{d\tilde{t}_{s}} = F_{3}(y_{8}), \end{aligned}$$

3b $$\begin{aligned}& \delta \frac{d y_{8}}{d\tilde{t}_{s}} = F_{8}(v_{0}, v_{2}, v_{3}, y_{8}), \end{aligned}$$

3c $$\begin{aligned}& \frac{d v_{0}}{d\tilde{t}_{s}} = F_{0}(y_{5}), \end{aligned}$$

3d $$\begin{aligned}& \frac{d y_{5}}{d\tilde{t}_{s}} = F_{5}(v_{0}, v_{1}, v_{2}, v_{3}, y_{5}), \end{aligned}$$

3e $$\begin{aligned}& \frac{d v_{1}}{d\tilde{t}_{s}} = F_{1}(y_{6}), \end{aligned}$$

3f $$\begin{aligned}& \frac{d y_{6}}{d\tilde{t}_{s}} = F_{6}(v_{0}, v_{1}, y_{6}), \end{aligned}$$

3g $$\begin{aligned}& \frac{d v_{2}}{d\tilde{t}_{s}} = \varepsilon F_{2}(y_{7}), \end{aligned}$$

3h $$\begin{aligned}& \frac{d y_{7}}{d\tilde{t}_{s}} = \varepsilon F_{7}(v_{0}, v_{2}, y_{7}), \end{aligned}$$

where the functions $F_{i}(\cdot)$ are as defined in (2a)–(2h). The *super-slow system* is obtained by defining $\tilde{t}_{ss} = \varepsilon \tilde{t}_{s} = \varepsilon \delta \tilde{t}$:

4a $$\begin{aligned}& \delta \varepsilon \frac{d v_{3}}{d\tilde{t}_{ss}} = F_{3}(y_{8}), \end{aligned}$$

4b $$\begin{aligned}& \delta \varepsilon \frac{d y_{8}}{d\tilde{t}_{ss}} = F_{8}(v_{0}, v_{2}, v_{3}, y_{8}), \end{aligned}$$

4c $$\begin{aligned}& \varepsilon \frac{d v_{0}}{d\tilde{t}_{ss}} = F_{0}(y_{5}), \end{aligned}$$

4d $$\begin{aligned}& \varepsilon \frac{d y_{5}}{d\tilde{t}_{ss}} = F_{5}(v_{0}, v_{1}, v_{2}, v_{3}, y_{5}), \end{aligned}$$

4e $$\begin{aligned}& \varepsilon \frac{d v_{1}}{d\tilde{t}_{ss}} = F_{1}(y_{6}), \end{aligned}$$

4f $$\begin{aligned}& \varepsilon \frac{d y_{6}}{d\tilde{t}_{ss}} = F_{6}(v_{0}, v_{1}, y_{6}), \end{aligned}$$

4g $$\begin{aligned}& \frac{d v_{2}}{d\tilde{t}_{ss}} = F_{2}(y_{7}), \end{aligned}$$

4h $$\begin{aligned}& \frac{d y_{7}}{d\tilde{t}_{ss}} = F_{7}(v_{0}, v_{2}, y_{7}). \end{aligned}$$

Systems (2a)–(2h), (3a)–(3h) and (4a)–(4h) describe different dynamics in the singular limits $\varepsilon \to 0$ and/or $\delta \to 0$, although they are equivalent for $\varepsilon \neq 0$ and $\delta \neq 0$. Letting $\delta \to 0$ in (2a)–(2h) yields the *fast layer problem* (2a)–(2b) which describes the dynamics of the fast variables $(v_{3}, y_{8})$ for fixed values of the slow ($v_{0}$) and super-slow ($v_{2}$) variables. The critical manifold is defined by the equilibrium points of the fast layer problem, that is,

5 $$ S^{0} = \bigl\{ (v_{3},y_{8},v_{0},y_{5},v_{1},y_{6} v_{2},y_{7}) \in \mathbb{R}^{8} \mid G S[C_{5} \tau _{a} v_{0} - C_{6} \tau _{b} v_{2} ] - v_{3} =0\bigr\} , $$

which is eventually in the $(y_{8}=0)$-space. Since the eigenvalues of the Jacobian matrix of the fast layer problem defined by (2a)–(2b) with respect to $(v_{3}, y_{8})$ are $\lambda _{1,2} = -1$, the 6-dimensional critical manifold $S^{0}$ is normally hyperbolic and stable, thus, it is perturbed to local slow manifolds for sufficiently small $\delta >0$. Therefore, the fast dynamics can be approximated by slow dynamics as suggested by the Fenichel theorem [51].

Setting $\delta \to 0$ in (3a)–(3h) gives an algebraic-differential *slow reduced problem*,

6a $$\begin{aligned}& 0 = F_{3}(y_{8}), \end{aligned}$$

6b $$\begin{aligned}& 0 = F_{8}(v_{0}, v_{2}, v_{3}, y_{8}), \end{aligned}$$

6c $$\begin{aligned}& \frac{d v_{0}}{d\tilde{t}_{s}} = F_{0}(y_{5}), \end{aligned}$$

6d $$\begin{aligned}& \frac{d y_{5}}{d\tilde{t}_{s}} = F_{5}(v_{0}, v_{1}, v_{2}, v_{3}, y_{5}), \end{aligned}$$

6e $$\begin{aligned}& \frac{d v_{1}}{d\tilde{t}_{s}} = F_{1}(y_{6}), \end{aligned}$$

6f $$\begin{aligned}& \frac{d y_{6}}{d\tilde{t}_{s}} = F_{6}(v_{0}, v_{1}, y_{6}), \end{aligned}$$

6g $$\begin{aligned}& \frac{d v_{2}}{d\tilde{t}_{s}} = \varepsilon F_{2}(y_{7}), \end{aligned}$$

6h $$\begin{aligned}& \frac{d y_{7}}{d\tilde{t}_{s}} = \varepsilon F_{7}(v_{0}, v_{2}, y_{7}), \end{aligned}$$

which describes the slow dynamics restricted to $S^{0}$. System (6a)–(6h) is a two-time-scale system of 4 slow/2 super-slow variables with *ε* being the time-scaling parameter. The equilibria of the *slow layer problem* in the $\varepsilon \to 0$ limit defines the *super-slow manifold* $L^{0}$, which is reduced to

7 $$\begin{aligned} L^{0} = & \bigl\{ (v_{3},y_{8},v_{0},y_{5},v_{1},y_{6} v_{2},y_{7}) \in S^{0} \mid \\ & A S\bigl[ A \tau _{a} p+C_{2} \tau _{a} A S[C_{1} \tau _{a} v_{0} ]- C_{4} \tau _{b} v_{2}-C_{7} \tau _{g} v_{3} \bigr]-v_{0}=0\bigr\} , \end{aligned}$$

and restricted to $S^{0}$ by the algebraic condition $v_{3} = G S[C_{5} \tau _{a} v_{0} - C_{6} \tau _{b} v_{2}] = \mathcal{K}(v_{0}, v_{2})$ in (6a)–(6b). The super-slow dynamics restricted to the 2-dimensional manifold $L^{0}$, hence to $S^{0}$, are given by the super-slow reduced system in the $\varepsilon \to 0$ limit of (4a)–(4h).

In order to investigate the super-slow flow on $L^{0}$, we consider the two-time-scale system (6a)–(6h) with the fast variable $v_{3}$ on $S^{0}$, i.e. $v_{3} = \mathcal{K}(v_{0},v_{2})$, and rewrite the slow reduced system (6a)–(6h) as

8a $$\begin{aligned}& \frac{d v_{0}}{d\tilde{t}_{s}} = F_{0}(y_{5}), \end{aligned}$$

8b $$\begin{aligned}& \frac{d y_{5}}{d\tilde{t}_{s}} = F_{5}\bigl(v_{0}, v_{1}, v_{2}, \mathcal{K}(v_{0},v_{2}), y_{5} \bigr), \end{aligned}$$

8c $$\begin{aligned}& \frac{d v_{1}}{d\tilde{t}_{s}} = F_{1}(y_{6}), \end{aligned}$$

8d $$\begin{aligned}& \frac{d y_{6}}{d\tilde{t}_{s}} = F_{6}(v_{0}, v_{1}, y_{6}), \end{aligned}$$

8e $$\begin{aligned}& \frac{d v_{2}}{d\tilde{t}_{s}} = \varepsilon F_{2}(y_{7}), \end{aligned}$$

8f $$\begin{aligned}& \frac{d y_{7}}{d\tilde{t}_{s}} = \varepsilon F_{7}(v_{0}, v_{2}, y_{7}). \end{aligned}$$

Applying the time-scaling $\tilde{\tau }_{s} = \varepsilon \tilde{t}_{s}$ and taking the singular limit $\varepsilon \to 0$ give the algebraic-differential system

9a $$\begin{aligned}& 0 = F_{0}(y_{5}), \end{aligned}$$

9b $$\begin{aligned}& 0 = F_{5}\bigl(v_{0}, v_{1}, v_{2}, \mathcal{K}(v_{0},v_{2}), y_{5}\bigr), \end{aligned}$$

9c $$\begin{aligned}& 0 = F_{1}(y_{6}), \end{aligned}$$

9d $$\begin{aligned}& 0 = F_{6}(v_{0}, v_{1}, y_{6}), \end{aligned}$$

9e $$\begin{aligned}& \frac{d v_{2}}{d\tilde{\tau }_{s}} = F_{2}(y_{7}), \end{aligned}$$

9f $$\begin{aligned}& \frac{d y_{7}}{d\tilde{\tau }_{s}} = F_{7}(v_{0}, v_{2}, y_{7}). \end{aligned}$$

The algebraic conditions (9a)–(9d) define the ‘critical manifold’ of (8a)–(8f) which is equivalent to $L^{0}$ given by (7). Notice that $L^{0}$ is restricted in the zero plane of the $(y_{5}, y_{6})$-space. Assuming that $v_{2}$ is some function of $v_{0}$ on $L^{0}$, i.e. $v_{2} = \mathcal{M}(v_{0})$, the fold points on $L^{0}$ are defined by

10 $$ \mathcal{F} = \biggl\{ (v_{0}, v_{1}, v_{2}, v_{3}, y_{5}, y_{6}, y_{7}, y_{8}) \in L^{0} \Bigm| v_{2} = \mathcal{M}(v_{0}), \frac{\partial \mathcal{M}(v_{0})}{\partial v_{0}} = 0 \biggr\} . $$

**Figure 2:**
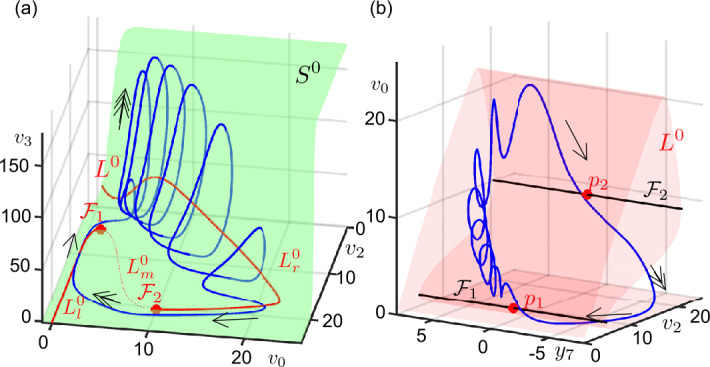
Critical manifold, slow manifold and folded singular points. (**a**) Critical manifold $S^{0}$ (green surface), super-slow manifold $L^{0}$ (red curve) and a bursting orbit in the $(v_{0}, v_{2}, v_{3})$-space. The curve $L^{0}$ is divided into three branches at $\mathcal{F}_{1}$ and $\mathcal{F}_{2}$ (red dots) where it changes stability. The middle branch of the $L^{0}$ ($L^{0}_{m}$) curve between $\mathcal{F}_{1}$ and $\mathcal{F}_{2}$ is unstable (dashed). The stable left-hand side branch ($L^{0}_{l}$), $\mathcal{F}_{1}$, $L^{0}_{m}$ and $\mathcal{F}_{2}$ are entirely on the almost horizontal part of $S^{0}$ (approximately on the $(v_{3} \approx 0)$-plane). The stable right-hand side branch of $L^{0}$ ($L^{0}_{r}$) on expands both on the horizontal and vertical parts of $S^{0}$. Arrows indicate the corresponding time-scale (single-headed for super-slow, double-headed for slow dynamics). (**b**) Super-slow manifold $L^{0}$ (red surface), fold curves $\mathcal{F}_{1,2}$ (black lines) and folded singular points $p_{1,2}$ (red dots) in the $(y_{7}, v_{2}, v_{0})$-space. Arrows indicate the corresponding time-scale

Figure 2a shows $S^{0}$ and $L^{0}$ in the $(v_{0}, v_{2}, v_{3})$-space, and Fig. 2b $L^{0}$ in the $(y_{7}, v_{2}, v_{0})$-space. The super-slow manifold $L^{0}$ expands between the lower horizontal and vertical planes of $S^{0}$. The part of curve $L^{0}$ on the lower horizontal plane of $S^{0}$ is folded with respect to $v_{2}$ at along the fold curves $\mathcal{F}_{1}$ and $\mathcal{F}_{2}$ defined by (10), i.e. $\mathcal{F} = \mathcal{F}_{1} \cup \mathcal{F}_{2}$. In this projection, the 1-D fold curves divide $L^{0}$ into two stable (left-hand side $L^{0}_{l}$ and right-hand side $L^{0}_{r}$) and one unstable (middle $L^{0}_{m}$) branches on the $(v_{0}, v_{2}, v_{3})$-space. We also verify that four eigenvalues of $L^{0}$ (two real and two complex conjugate) have negative real parts along the stable parts of $L^{0}$. One of the real eigenvalues changes sign along $\mathcal{F}_{1,2}$, hence the unstable middle branch is of saddle type. Along the stable and unstable branches $L^{0}$ is normally hyperbolic, so $L^{0}$ is perturbed to local super-slow manifolds for small values of $\varepsilon > 0$ within (6a)–(6h); see the extension of Fenichel theory for systems with more than two time-scales [52]. On the other hand, the dynamics near the non-hyperbolic fold curves $\mathcal{F}_{1,2}$ should be investigated by using the elements of GSPT.

As being the usual strategy, we consider the desingularized version of the super-slow dynamics on $L^{0}$ is given by the *desingularized slow reduced system* (DSRS), reading

11a $$\begin{aligned}& \frac{d v_{0}}{d\hat{\tau }_{s}} = -y_{7}, \end{aligned}$$

11b $$\begin{aligned}& \frac{d y_{7}}{d\hat{\tau }_{s}} = - \frac{\partial \mathcal{M}(v_{0})}{\partial v_{0}} F_{7}\bigl(v_{0}, \mathcal{M}(v_{0}), y_{7}\bigr), \end{aligned}$$

where $\tilde{\tau }_{s} = \frac{\partial \mathcal{M}(v_{0})}{\partial v_{0}} \hat{\tau }_{s}$. The equilibria of (11a)–(11b) on the fold set $\mathcal{F}$ are located at $(v_{0}^{p1}, y_{7}^{p1})=(1.2343,0)$ and $(v_{0}^{p2}, y_{7}^{p2})=(9.9976,0)$ for the parameter set given in Table 1. These equilibrium points, which are not generally the true equilibria of (8a)–(8f), are related to the folded singularities of (8a)–(8f), hence of (2a)–(2h). On the other hand, equilibrium points $(v_{0}^{F_{7}}, y_{7}^{F_{7}})$, i.e. $F_{7}(v_{0}^{F_{7}}, y_{7}^{F_{7}}) = 0$, are ordinary singularities since they are also equilibria of (8a)–(8f), hence of (2a)–(2h). Figure 2b shows $L^{0}$, fold curves $\mathcal{F}_{1,2}$ and folded singular points $p_{1,2}$ in the $(y_{7}, v_{2}, v_{0})$-space.

Stability of the equilibrium points of the desingularized (slow) reduced system on the fold set determines the type of the folded singularities of the original system. Classification of these equilibrium points is based on the linear stability analysis. When the desingularized (slow) reduced system is planar, this analysis can be done using the trace and the determinant of the Jacobian matrix at the fold equilibrium. If both are different from zero, the fold equilibrium can be a folded saddle, a folded node or a folded focus. If the determinant equals zero but not the trace, then the desingularized flow has a degenerate equilibrium point, which is a folded saddle-node. A folded saddle-node is either related to a saddle-node bifurcation of the folded equilibria or a transcritical bifurcation of a folded equilibrium with an ordinary equilibrium. The latter case refers to the *folded saddle-node type II* (FSN II) singularity [53, 54], where a folded node becomes a folded saddle and a regular saddle becomes a regular node. The original system exhibits a singular Hopf bifurcation close to a FSN II singularity [55, 56].

The Jacobian matrix of (11a)–(11b) has the following general form:

12 $$\begin{aligned} J = \begin{bmatrix} 0 & -1 \\ -\frac{\partial ^{2} \mathcal{M}(v_{0}^{*})}{\partial v_{0}^{2}} F_{7}(v_{0}^{*}, y_{7}^{*}) - \frac{\partial \mathcal{M}(v_{0}^{*})}{\partial v_{0}} \frac{\partial {F_{7}(v_{0}^{*}, y_{7}^{*})}}{\partial v_{0}} & 2 \frac{\partial \mathcal{M}(v_{0}^{*})}{\partial v_{0}} \end{bmatrix}, \end{aligned}$$

where $(v_{0}^{*}, y_{7}^{*})$ stands for the equilibrium point of interest. Since on the folded equilibria $2\frac{\partial \mathcal{M}(v_{0}^{p1, p2})}{\partial v_{0}}= 0$, the trace and determinant of (12) on the folded equilibria read

13 $$\begin{aligned} \textbf{tr}\bigl(J^{p1, p2}\bigr) = 0, \qquad \textbf{det} \bigl(J^{p1, p2}\bigr) = - \frac{\partial ^{2} \mathcal{M}(v_{0}^{p1, p2})}{\partial v_{0}^{2}} F_{7} \bigl(v_{0}^{p1, p2}, y_{7}^{p1, p2}\bigr). \end{aligned}$$

The trace and determinant of (12) on the regular equilibria read

14 $$\begin{aligned} \textbf{tr}\bigl(J^{F_{7}}\bigr) = 2 \frac{\partial \mathcal{M}(v_{0}^{F_{7}})}{\partial v_{0}}, \qquad \textbf{det}\bigl(J^{F_{7}}\bigr) = - \frac{\partial \mathcal{M}(v_{0}^{F_{7}})}{\partial v_{0}} \frac{\partial {F_{7}(v_{0}^{F_{7}}, y_{7}^{F_{7}})}}{\partial v_{0}}. \end{aligned}$$

Notice that the generic folded singularity condition is violated due to the fact that $\frac{\partial (\mathcal{F}_{v_{2}}\dot{v}_{2} +\mathcal{F}_{y_{7}}\dot{y}_{7} )}{\partial v_{0}} = 0$ in (12), and $\textbf{tr}(J^{p1, p2}) = 0$ in (13). Therefore, the folded singularities determined by (11a)–(11b) are not generic and a folded equilibrium is one of the following types: a saddle for $\textbf{det}(J^{p1, p2})<0$, a center for $\textbf{det}(J^{p1, p2})>0$, a nilpotent for $\textbf{det}(J^{p1, p2}) = 0$. The latter degenerate type corresponds to a point in the parameter space at which a folded singularity and a regular singularity meet, i.e. $\textbf{tr}(J^{F_{7}}) = 0$ and $\textbf{det}(J^{F_{7}}) = 0$ in (14). Consequently, the equilibrium points of (11a)–(11b) related to the folded and regular singularities undergo *degenerate* transcritical bifurcations where (12) has two zero-eigenvalues.

**Figure 3:**
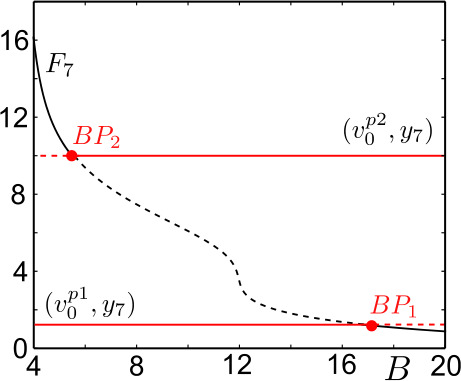
Bifurcation diagram of (11a)–(11b) with respect to *B*. Equilibrium points $(v_{0}^{p1}, y_{7}^{p1})$ lie on the lower red horizontal line $(v_{0}^{p1}, y_{7})$, and $(v_{0}^{p2}, y_{7}^{p2})$ on the upper red horizontal line $(v_{0}^{p2}, y_{7}^{p2})$. Dashed parts of the red lines represent saddle type, solid parts represent center type solutions. The true equilibrium points line on the black curve, $F_{7}$. Dashed part of the black curve represents saddle type, solid parts stable focus type solutions. The saddle type equilibrium points along $(v_{0}^{p1}, y_{7}^{p1})$ and $(v_{0}^{p2}, y_{7}^{p2})$ change to center at the intersections with $F_{7}$ at $BP_{1}$ and $BP_{2}$, respectively

Figure 3 shows the bifurcation diagram of (11a)–(11b) with respect to *B* in the region of interest. Two straight lines of the equilibria $(v_{0}^{p1}, y_{7}^{p1})$ and $(v_{0}^{p2}, y_{7}^{p2})$ intersect with the regular equilibria curve $F_{7}(v_{0}, y_{7})$ at two bifurcation points, $BP_{1}$ at $B_{BP1} \approx 16.7817$ and $BP_{2}$ at $B_{BP2} \approx 5.4817$, which are degenerate transcritical bifurcations. For $B< B_{BP1}$, the equilibrium $(v_{0}^{p1}, y_{7}^{p1})$ is a center with two complex conjugate eigenvalues. After the bifurcation at $BP_{1}$, $(v_{0}^{p1}, y_{7}^{p1})$ becomes a saddle. Consequently, the system (8a)–(8f) (and (2a)–(2h)) has a folded-saddle singularity near $p_{1}$ for $B > B_{BP1}$. The equilibrium $(v_{0}^{p2},y_{7}^{p2})$ is of a saddle type for $B < B_{BP2}$ and becomes a center with two complex conjugate eigenvalues at $B = B_{BP2}$. Hence, the system (8a)–(8f) (and (2a)–(2h)) has a folded-saddle singularity near $p_{2}$ for $B < B_{BP2}$. Finally, in a neighborhood of $BP_{1}$, the equilibrium points along the $F_{7}(v_{0}, y_{7})$ curve are of saddle type for $B < B_{BP1}$ and stable focus for $B > B_{BP1}$. Similarly, in a neighborhood of $BP_{2}$, the equilibrium points along the $F_{7}(v_{0}, y_{7})$ curve are of stable focus type for $B < B_{BP2}$ and of saddle type for $B > B_{BP2}$.

**Figure 4:**
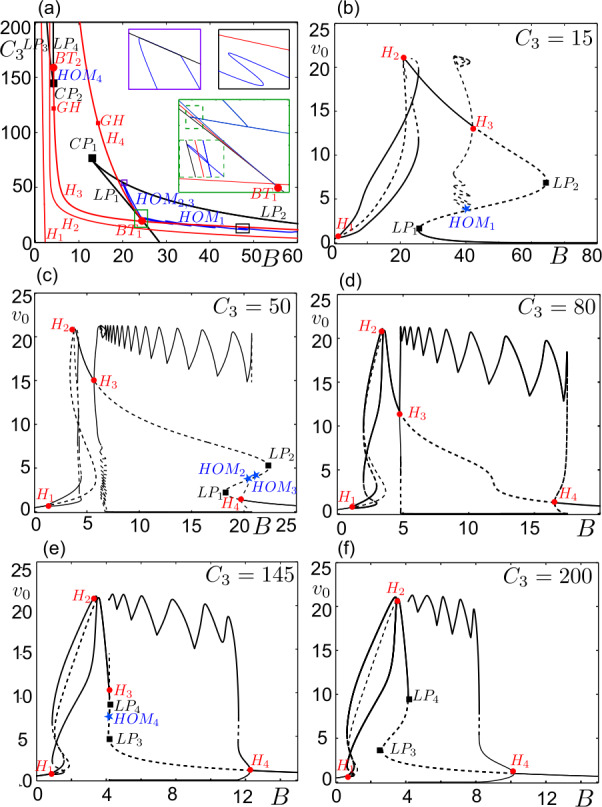
Bifurcation diagrams of (2a)–(2h) with as a function of $(B, C_{3})$. (**a**) Bifurcation diagram of (2a)–(2h) on the $(B, C_{3})$ plane. Curves are named, respectively, after the limit point (LP, black curves), Hopf (H, red curves) and homoclinic (HOM, blue curves) bifurcations in panels (**b**–**f**). Only the LP bifurcations interacting with canard solutions are plotted. Black squares indicate cusp (CP), red circles indicate Bogdanov–Takens (BT) and red squares indicate generalized Hopf (GF) bifurcations. The regions marked by black, green and purple boxes are zoomed in black, green and purple framed insets. The region where the homoclinic curve tips to the $LP_{1}$ is zoomed inside the green inset. (**b**–**f**) Bifurcation diagrams of (2a)–(2h) as a function of *B* for different values of $C_{3}$. The limit point bifurcations of interest are marked by black squares, Hopf bifurcations by red circles, and homoclinic connections by blue stars. Stable and unstable solutions are represented by continuous and dashed curves, respectively. Along the curves of equilibrium points, (2a)–(2h) undergoes four Hopf bifurcations ($H_{1,2,3,4}$) for $C_{3}=\{50,80,145\}$ (**c**, **d**, **e**) and three Hopf bifurcations ($H_{1,2,3}$) for $C_{3}=15$ (**b**) and ($H_{1,2,4}$) for $C_{3}=200$ (**f**)

As mentioned above, a generic transcritical bifurcation of regular and folded singularities is related to a FSN II singularity. In our case, a folded saddle becomes a folded center and a stable focus becomes a saddle at the *degenerate* transcritical bifurcation points $BP_{1}$ and $BP_{2}$. Furthermore, system (2a)–(2h) can undergo the (singular) Hopf bifurcations close to ${BP_{2}}$ and ${BP_{1}}$ in the parameter space (see Fig. 4d), as it will be detailed in the following sections. Hence, the interaction of regular and non-generic folded singularities can be referred as a *degenerate FSN II singularity*. A degenerate FSN II singularity in (2a)–(2h) stems from the structure of the NMM, which is defined as a second-order system that violates the generic folded singularity condition $\textbf{tr}(J)\neq 0$.

Folded singularities can lead to canard solutions in the original system. In a planar slow–fast system, a singular Hopf bifurcation can occur near a folded singularity, which is then called a *canard point*. In such case, the amplitude of the periodic orbits bifurcated at the singular Hopf point increases stiffly in a narrow interval of the parameter (scaled by the time-scale separation parameter) that controls the transition from small amplitude to relaxation oscillations [57]. This phenomenon is known as *canard explosion* [26, 58]. A canard-explosive branch hosts small canards following the unstable branch of the critical manifold and one stable branch (so-called *canard-without-head solutions*), large canards following the unstable branch of the critical manifold and two stable branches (so-called *canard-with-head solutions*), and a *maximal canard solution* that follows the longest the repelling branch. In planar multiple time-scale systems, canard solutions are tightly connected to excitability and firing thresholds [30, 31]. In higher dimensional multiple time-scale systems with at least two slow variables, the folded-singularities are generic, hence they are robust to small parameter perturbations, and canard solutions associated with folded singularities connect stable and unstable branches of a folded critical manifold [36, 53, 59–61]. Canards of folded node and FSN II singularities support mixed mode oscillations [27, 36, 44]. FSN II singularities have been identified in neuronal models where the exchange from an excitable to a relaxation oscillatory state is accompanied by subthreshold oscillations [24, 28, 42, 62]. Folded-saddle canards have been shown to be sculpting firing threshold manifolds, as well [33, 34, 63–65].

In our problem, the critical manifold $S^{0}$ (5) is hyperbolic, whereas the super-slow manifold $L^{0}$ (8a)–(8f) has a folded structure. Thus, the critical transitions occur mainly in the 6-dimensional reduced system given by (8a)–(8f). As the analysis above have shown, (8a)–(8f) has degenerate folded singularities along the fold curve at $p_{1}$ and $p_{2}$. Notice that, since the system has neither a folded node nor a FSN II, small amplitude oscillations do not exist near $p_{1}$ or $p_{2}$. But the folded saddle, degenerate FSN II and singular Hopf bifurcations can lead to canard solutions governing the critical transitions in (8a)–(8f) (hence in (2a)–(2h)). On the other hand, the bursting behavior cannot be captured by (8a)–(8f) because (8a)–(8f) is restricted in the critical manifold $S^{0}$, whereas the fast oscillations of the bursting orbits leave off $S^{0}$. So the bursting solutions exist in the full system (2a)–(2h) (see [48] for a detailed analysis of the bursting solutions). As a result, our problem yields both three and two time-scaled behaviors. In the next section, we investigate canard dynamics near $p_{1}$ and $p_{2}$.

## Multiple time-scale oscillations and canard transitions

Transitions near the folded singularities of (2a)–(2h) which lead to canard solutions depend on the system parameters. The reader may refer to Table 1 for the parameter values, unless otherwise stated. The connectivity strength from the pyramidal cell population on the subpopulation of the SOM+ interneurons, $C_{3}$, and their synaptic gain, *B*, appear as two crucial parameters controlling the transitions by affecting the curve $F_{7}$ in (11a)–(11b) (see Fig. 3). Figure 4a shows a 2-parameter bifurcation diagram in the plane $(B, C_{3})$. Depending on $C_{3}$, system (2a)–(2h) undergoes several Hopf bifurcations as a function of *B*. The first two Hopf bifurcations, $H_{1}$ and $H_{2}$, yield harmonic oscillations, whereas the periodic branches appearing at $H_{3}$ and/or $H_{4}$ connect to multiple time-scale oscillations. Under the variations in $(B, C_{3})$, $H_{1}$ and $H_{2}$ persist; and the emerging periodic orbits do not change qualitatively. On the other hand, $H_{3}$ and $H_{4}$ undergo Bogdanov–Takens (BT) bifurcations $BT_{1,2}$ and the corresponding periodic branches vary qualitatively. The periodic orbits emerging at $H_{3}$ and $H_{4}$ can end on homoclinic connections, namely $HOM_{1,2,3,4}$.

Figures 4b–f exemplify qualitative variations in (2a)–(2h) as a function of *B* for different values of $C_{3}$. For $C_{3} < C_{3, BT1}\approx 18.9$, the system undergoes three Hopf bifurcations, for instance in Fig. 4b for $C_{3}=15$. The branch of periodic solutions starting at $H_{3}$ terminates at a homoclinic connection, $HOM_{1}$. As $C_{3}$ increases, $HOM_{1}$ and $LP_{1}$ get closer while the amplitude and the number of spikes of the periodic orbits increase. The spike adding occurs as the $HOM_{1}$ curve folds back and forth in the $(B, C_{3})$-space (see the black framed inset in Fig. 4a for an example folding). At $C_{3} = C_{3, BT1}$ another Hopf bifurcation, $H_{4}$, appears yielding a new branch of periodic orbits making a second homoclinic connection, $HOM_{2}$ (green framed inset in Fig. 4a). Consequently, $HOM_{1}$ and $HOM_{2}$ points coexist in a narrow range of $(B, C_{3})$. The $HOM_{1}$ curve touches the $LP_{1}$ curve at $(B, C_{3}) \approx (23.98, 22.43)$, folds back and continues in the parameter space, which then we name as $HOM_{3}$ curve (dashed zone in the green framed inset in Fig. 4a). The curves $HOM_{1}$ and $HOM_{3}$ stay very close to each other in $(23.98< B<24.46, 21.94<C_{3}<22.43)$, before $HOM_{3}$ bends in the $C_{3}$ direction at $(B, C_{3}) \approx (24.46, 22.43)$. As it happens, the branch of periodic orbits curls below $LP_{1}$ in the *B*-space and eventually connects to $HOM_{3}$. With increasing $C_{3}$, this branch of periodic orbits advances further towards the stable equilibrium points while introducing a region of multi-attractors of nodes, saddles, unstable small oscillations and stable large amplitude bursting oscillations (see Fig. 4c for $C_{3} = 50$, dynamics will be detailed in Sect. 3.2). Concurrently, $H_{4}$ moves away from $LP_{1}$ and $HOM_{2,3}$ approach $LP_{2}$. In $(20.09< B<20.26, 54.08<C_{3}<54.43)$, $HOM_{2}$ and $HOM_{3}$ curves are connected by a section that is parallel to the $LP_{2}$ curve (purple framed inset in Fig. 4a). For $C_{3}>54.43$, the branch of periodic orbits initiated at $H_{4}$ connects to the branch of large amplitude multiple time-scale oscillations (Fig. 4d).

System (2a)–(2h) does not have any LPs between $H_{3}$ and $H_{4}$ for $C_{3, CP1}\leq C_{3} \leq C_{3, CP2}$. At $C_{3} = C_{3, CP2}\approx 141.4$, as the lower branch of equilibrium points curls below $H_{3}$, the connection between the large amplitude orbits and $H_{3}$ is broken up on a saddle-saddle homoclinic bifurcation (the equilibrium points in a neighborhood of $LP_{3}$ for $B \geq B_{LP3}$ are saddles [66, 67]). As a consequence, the branch of periodic orbits starting from $H_{3}$ terminates on a homoclinic connection, $HOM_{4}$ (for $C_{3}=145$ in Fig. 4e). This homoclinic connection remains until $H_{3}$ disappears at $C_{3} = C_{3, BT2} \approx 157$. Beyond $C_{3} > C_{3, BT2}$, the large amplitude bursting orbits introduced by $H_{4}$ terminate on a saddle-node homoclinic connection (for instance at $C_{3} =200$ in Fig. 4f).

The Hopf bifurcations $H_{3}$ and $H_{4}$ occur close to the folded singularities $p_{2}$ and $p_{1}$, respectively. System (2a)–(2h) can yield canard solutions close to these points in the parameter space of *B*, such as $B\approx B_{H3}$ and $B\approx B_{H4}$. In the following section, we will show the canard-mediated transition from sinusoidal oscillations initiated by $H_{3}$ to large amplitude bursting/relaxation type solutions. Subsequently, Sect. 3.2 will detail the canard dynamics and related excitability near $H_{4}$, in particular, the type-I excitability for $C_{3} = 50$ and type-II excitability for $C_{3} = 80$.

### Canard-mediated transitions between sinusoidal and multiple time-scale oscillations

**Figure 5:**
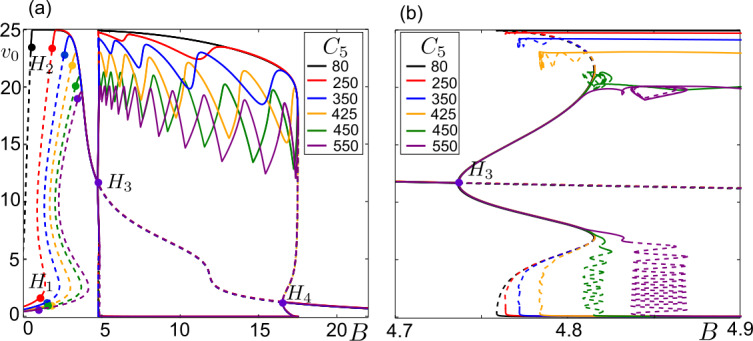
Variation of large amplitude solutions with respect to $(B, C_{5})$. (**a**) Bifurcation diagrams of (2a)–(2h) with respect to *B* for $C_{3}=80$ and different values of $C_{5}$. Curves and Hopf bifurcations ($H_{1,2,3,4}$, dots) are colored with respect to the color codes of $C_{5}$ values. Stable and unstable solutions are represented by continuous and dashed curves, respectively. For the sake of simplicity, the periodic solutions between $H_{1}$ and $H_{2}$ are not shown. (**b**) Zoom into the region of transitions between sinusoidal and large amplitude multiple-time-scale solutions in $B\in [4.7,4.9]$

Köksal Ersöz et al. [48] have realized that the number of spikes of a bursting solution of (2a)–(2h) depends on the amount of the PSP received by the PV+ interneuron subpopulation, hence on the EPSP coming from the pyramidal cell subpopulation and the IPSP from the SOM+ interneurons. For instance, increasing the IPSP on the PV+ interneurons by increasing *B* decreases the number of spikes while driving the oscillations one peak to the next one in the parameter space (see Fig. 4c–f and 5). The connectivity constant from the pyramidal cell subpopulation to the PV+ interneuron subpopulation, $C_{5}$, directly scales the EPSP on this subpopulation, therefore determines the maximum number of fast spikes of the bursting oscillations, or more generally, the type of the multiple time-scale oscillations.

Figure 5 exemplifies how $C_{5}$ modulates the large amplitude oscillations between $H_{3}$ and $H_{4}$ on the bifurcation diagram of (2a)–(2h) for $C_{3} = 80$. Increasing $C_{5}$ decreases the amplitude of the oscillations, moves $H_{1}$ and $H_{2}$ slightly to the right, but does not affect considerably the locations $H_{3}$ and $H_{4}$ with respect to *B* (Fig. 5a). The supercritical Hopf bifurcation at $H_{3}$ yields a branch of sinusoidal periodic oscillations (Fig. 5b) that folds back and forth as *B* varies and enters in a regime of multiple time-scale periodic oscillations. These oscillations are of relaxation type for small values of $C_{5}$, and of bursting type for large values of $C_{5}$. Furthermore, the stable sinusoidal and multiple time-scale periodic oscillations can coexist depending on the values of $C_{5}$ (see Fig. 5b at $B \approx 4.8$).

**Figure 6:**
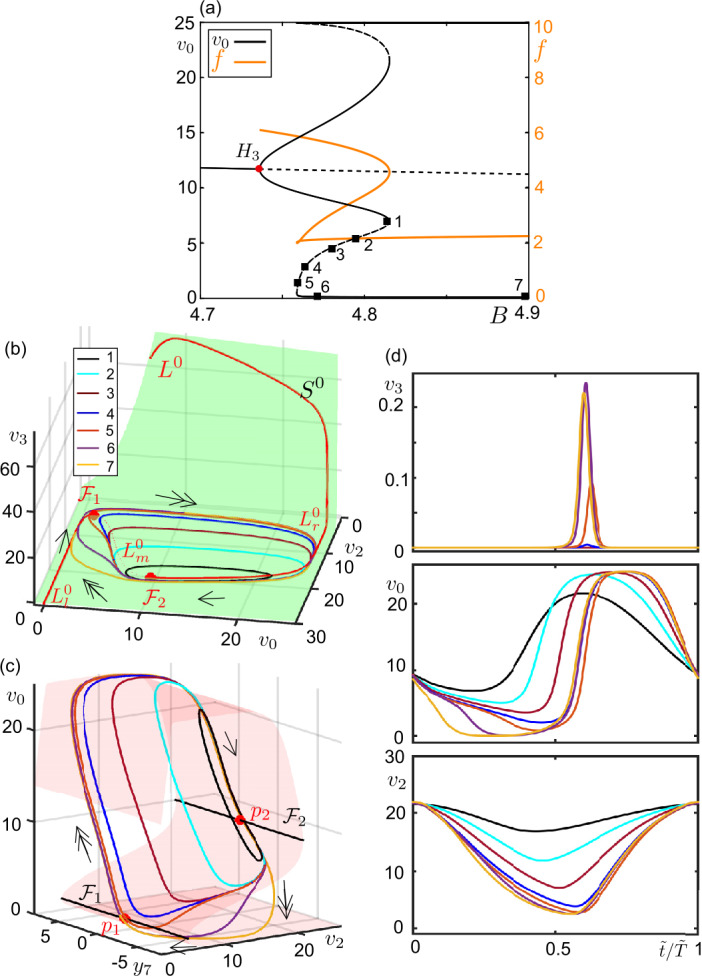
Example canard orbits along the transition from sinusoidal oscillations to relaxation oscillations. Zoom near the bifurcation diagram for $C_{3}= 80$, $C_{5}=80$ and $B \in [4.7, 4.9]$ (see Fig. 5a for the whole diagram). Continuous and dash curves represent stable and unstable solutions, respectively. Hopf ($H_{3}$, red dot) is marked on the diagram. Numbered orbits from 1-7 are given in panels (**b**–**d**). The orange curve traces the frequency of the oscillations emerging at $H_{3}$ The orange curve traces the frequency of the oscillations emerging at $H_{3}$. (**b**) Periodic orbits marked in panel (**a**), $L^{0}$ (red curve), fold curves $\mathcal{F}_{1,2}$ (red points) and the critical surface $S^{0}$ (green surface) are projected on the $(v_{0},v_{2},v_{3})$-space. Arrows indicate the corresponding time-scale (single-headed for super-slow, double-headed for slow dynamics). (**c**) Periodic orbits marked in panel (**a**), $L^{0}$ (red surface), fold curves $\mathcal{F}_{1,2}$ (black curves) and folded singular points $p_{1,2}$ (red dots) are projected on the $(y_{7},v_{2},v_{0})$-space. Arrows indicate the corresponding time-scale. (**d**) Time series of the periodic orbits on panels (**b**, **c**) with respective color codes. Period is normalized to 1 ($\tilde{t}/ \tilde{T} =1$, where *T̃* represents period of a cycle)

The form of the branch of periodic solutions between $H_{3}$ and $H_{4}$ in Fig. 5a indicates the type of the multiple time-scale oscillations for a certain parameter combination. For $C_{5} = 80$ (black diagram in Fig. 5) the smoothly decreasing amplitude of $v_{0}$ with *B* indicates that the corresponding orbits are of relaxation type (exemplified in Fig. 6). The horizontal zigzags along the upper part of the periodic branches obtained for greater values of $C_{5}$ indicate the presence of bursting solutions along these periodic branches and the number of their fast spikes. For instance, the 5 peaks that we count between $H_{3}$ and $H_{4}$ for $C_{5}=350$ (blue diagram in Fig. 5) signify that the maximum number of fast spikes for $C_{5}=350$ is 4. Such a bursting orbit is obtained for sufficiently small values of *B* ($B=5$, for instance). Then as *B* increases, the bursting orbits lose their fast spikes one by one through the peaks of the horizontal branch. They become relaxation cycles ($B=16$, for instance), before shrinking and disappearing via a subcritical Hopf bifurcation at $H_{4}$.

The periodic solutions connected to $H_{1}$ and $H_{2}$ do not interact with the singular fold points of $L^{0}$, $p_{1}$ and $p_{2}$, but the ones near $H_{3}$ and $H_{4}$ do because $H_{3}$ and $H_{4}$ take place close to the degenerate FSN II singularities on the fold curve $\mathcal{F}$. As a consequence, the multiple time-scale orbits emanating at singular Hopf bifurcations $H_{3}$ and $H_{4}$ can undergo canard explosion along which canard trajectories sculpt the periodic oscillations. Figure 6 shows example orbits along the periodic branch that follow $H_{3}$ for $C_{3}=80$ and $C_{5}=80$. As the periodic branch folds with respect to *B* and becomes unstable (Fig. 6a), the sinusoidal orbits of 4.5–6 Hz start to interact with $p_{2}$. In particular, they move along the unstable branch of $L^{0}$, $L^{0}_{m}$, before jumping back to the stable branch $L^{0}_{r}$. Hence, the periodic orbits become canard orbits (the 1st orbit). As *B* varies along the periodic branch in the parameter space, the canard orbits grow in amplitude along $L^{0}_{m}$ (the second, third and fourth orbits) until they stretch out between $\mathcal{F}_{1}$ and $\mathcal{F}_{2}$ (the fifth orbit). The canard orbits that oscillate between $L^{0}_{m}$ and $L^{0}_{r}$ can be interpreted as canard-without-head orbits, and the fifth orbit as the maximal canard since it has the largest period of the canard family of the periodic branch under consideration. Soon after the fifth orbit, the trajectories jump to the attracting branch $L^{0}_{l}$, get a shape of canard-with-head solutions, and become stable (the sixth orbit). As *B* increases, the relaxation cycles appear with parts exclusively following the attracting branches of $L^{0}$ and jumping close to the fold points.

As mentioned in the introduction, canard solutions play a fundamental role in separating different dynamical regimes. The unstable canard orbits in Fig. 6 (from the first to the fifth) appear as an other example of this phenomenon by accompanying the transition from sinusoidal oscillations to relaxation oscillations. For instance, sinusoidal oscillations and large amplitude canard-with-head cycles coexist for $B \in (4.75, 4.81)$ and the canard-without-head cycles form the boundary between them, as seen clearly in Fig. 6a. While increasing $C_{5}$ introduces bursting type of solutions, it can preserve the bistability between the bursting and sinusoidal oscillations, for example for $C_{5}=\{250, 350, 425\}$ in Fig. 5. Notice that with increasing $C_{5}$, the initially smooth branch of periodic orbits becomes steeper, gains vertical zigzags that move to the right along the *B*-axis, and the region of bistability decreases.

**Figure 7:**
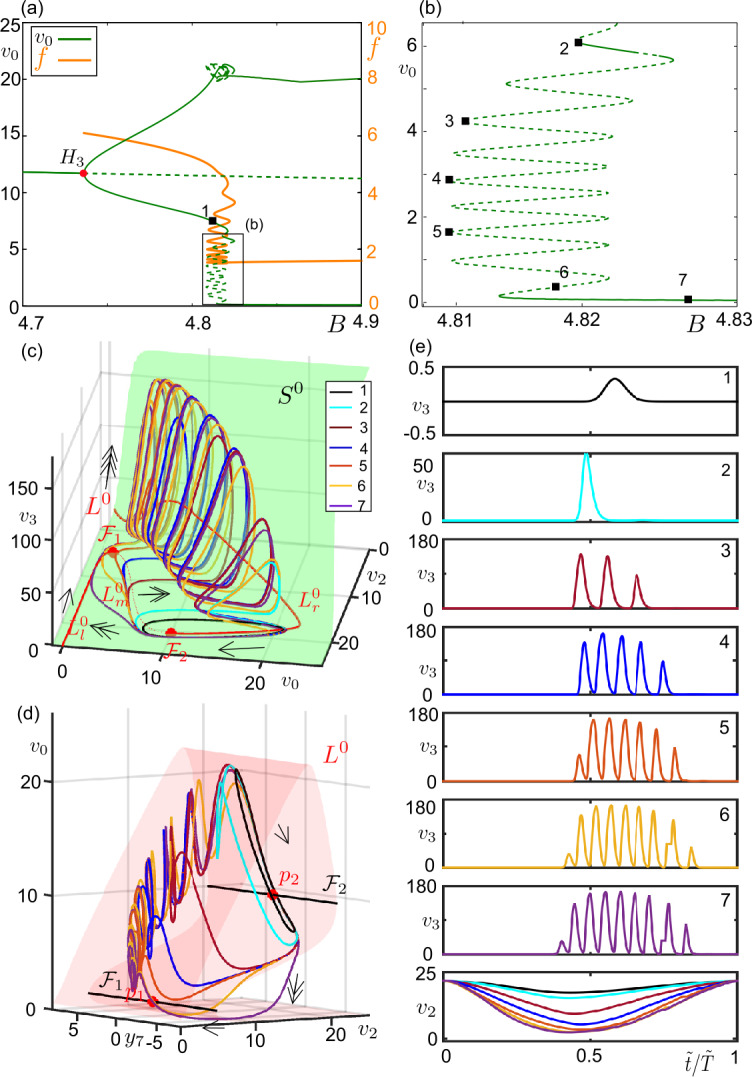
Example canard orbits along the transition from sinusoidal oscillations to bursting oscillations (**a**) Bifurcation diagram for $C_{3} = 80$, $C_{5}=450$ and $B \in [4.7, 4.9]$ (see Fig. 5a for the whole diagram). Stable and unstable solutions are represented by continuous and dashed curves, respectively. Hopf ($H_{3}$, red dot) is marked on the diagram. The rectangular region is zoomed in panel (**b**). Numbered orbits from 1-7 are given in panels (**c**–**d**). The orange curve traces the frequency of the oscillations emerging at $H_{3}$. (**c**) Periodic orbits marked in panels (**a**, **b**), $L^{0}$ (red curve), fold curves $\mathcal{F}_{1,2}$ (red points) and the critical surface $S^{0}$ (green surface) are projected on the $(v_{0},v_{2},v_{3})$-space. Arrows indicate the flow direction and its time-scale (single-head for super-slow, double-head for slow, triple-head for fast). (**d**) Periodic orbits marked in panel (**a**), $L^{0}$ (red surface), fold curves $\mathcal{F}_{1,2}$ (black curves) and folded singular points $p_{1,2}$ (red dots) are projected on the $(y_{7},v_{2},v_{0})$-space. Arrows indicate the corresponding time-scale. (**e**) Time series of the periodic orbits on panels are (**b**, **c**) shown with respective color codes. Period is normalized to 1 ($\tilde{t}/ \tilde{T} =1$, where *T̃* represents period of a cycle)

Figure 7 zooms into the region of canard orbits following the sinusoidal solutions of 4.8–6 Hz started at $H_{3}$ for $C_{5}=450$. As the stable sinusoidal oscillations grow in amplitude with increasing *B*, they start to interact with $p_{2}$ and to follow the bits of $L^{0}_{m}$ (the first orbit). Soon after, the orbits undergo a LP bifurcation (where the branch of the periodic orbits folds back at $B \approx 4.821$) and become unstable. As *B* varies in the parameter space along the periodic branch, the orbits moving along $L^{0}_{m}$ in the super-slow time-scale grow in amplitude and they start to interact with the vertical panel of $S^{0}$ as they jump to $L^{0}_{r}$ in the slow time-scale. So, the orbits become canard orbits.

As the part of the trajectory along $L^{0}_{m}$ grows in amplitude, the trajectory gets attracted by $L^{0}_{r}$ along the vertical panel of $S^{0}$ and it spirals around $L^{0}_{r}$ before landing on the horizontal plane of $S^{0}$. This interaction with the vertical panel of $S^{0}$ occurs in the fast time-scale, and eventually yields fast spikes, i.e., bursting-type canard oscillations. For instance, the second orbit in Fig. 7c and 7d has one fast spike. The number of spikes increases as the trajectory stays longer and longer along $L^{0}_{m}$ while *B* varies. More precisely, the number of spikes changes by one as we pass from one fold to another on the same side of along the snaking periodic branch with respect to *B* (Fig. 7b). For instance, solution-2 has 1 spike, solution-3, which is two fold below, has 3 spikes and so on. The spike adding continues until the canard orbits (analogous to canard-without-head orbits) expands between two folded singularities, hence till the occurrence of the maximal canard of the family (approximated by the sixth orbit). After the maximal canard, the canard cycles start to follow $L^{0}_{l}$ in the super-slow time-scale (analogous to canard-with-head orbits) and they become stable (the seventh orbit). The part of the trajectory along $L^{0}_{m}$ decreases as *B* increases further.

For $C_{3} = 80$, the system undergoes a complete canard explosion along the periodic branch following $H_{3}$ since it visits the whole canard family from small to large (Figs. 6 and 7). However, what we may observe for small values of $C_{3}$ is an incomplete canard explosion terminating at a homoclinic connection. For instance, for $C_{3} = 15$ (see Fig. 4b), the sinusoidal oscillations along the periodic branch initiated at $H_{3}$ change qualitatively by interacting with $L^{0}_{m}$ as *B* varies and we observe homoclinic canard-without-head orbits at $HOM_{1}$. The orbits terminating on $HOM_{3}$ are homoclinic canard-with-head orbits surrounded by stable large amplitude oscillations. Increasing $C_{3}$ completes the canard explosion and the system enters into an excitable regime which will be detailed in the following section.

### Canard-mediated transitions and excitability

According to Hodgkin’s [6] classification of neural excitability, type-I excitable neurons have continuous frequency-injected current curves, whereas type-II excitable neurons have discontinuous frequency-injected current curves. Rinzel and Ermentrout [68, 69] linked the type-I excitability to a SNIC bifurcation and the type-II excitability to a Hopf bifurcation. De Maesschalck and Wechselberger [29] explained the transition between the two excitability types via an intermediate regime of type-I excitability associated with a codimension-2 Bogdanov–Takens (BT) bifurcation in a planar system. They showed the existence of incomplete canard transitions in this transitory regime. Later on, transitions between the neuronal excitability types was shown to be induced by the inhibitory and excitatory autapse in the Morris-Lecar model [70]. Folded singularities and corresponding canard solutions in higher dimensional systems also have been shown to be shaping systems’ excitability properties [24, 28, 33, 34, 63–65].

System (2a)–(2h) can yield large amplitude oscillations in response to certain forms of stimulation (due to stochastic inputs, for instance) after being initiated from an equilibrium point for a *B* value close to $H_{4}$, $LP_{1}$ and $LP_{2}$ in Figs. 4c–4f. Hence, system (2a)–(2h) is excitable in these regions and the excitability properties of (2a)–(2h) determined by the parameter $C_{3}$ (see Fig. 4). Indeed, the local pictures in these regions are similar to the ones investigated in [29, 70]. In particular, system (2a)–(2h) is type-I excitable for $C_{3} \in (22.43, 54.43)$, basically between the homoclinic/saddle-saddle interactions near $LP_{1}$ and $LP_{2}$. In this parameter region, the large amplitude oscillations terminate on a homoclinic orbit for which the firing frequency is zero. System (2a)–(2h) is type-II excitable for $C_{3} > 54.43$ for which the termination is issued via a Hopf bifurcation. In both cases, canard solutions shape the resulting dynamics.

**Figure 8:**
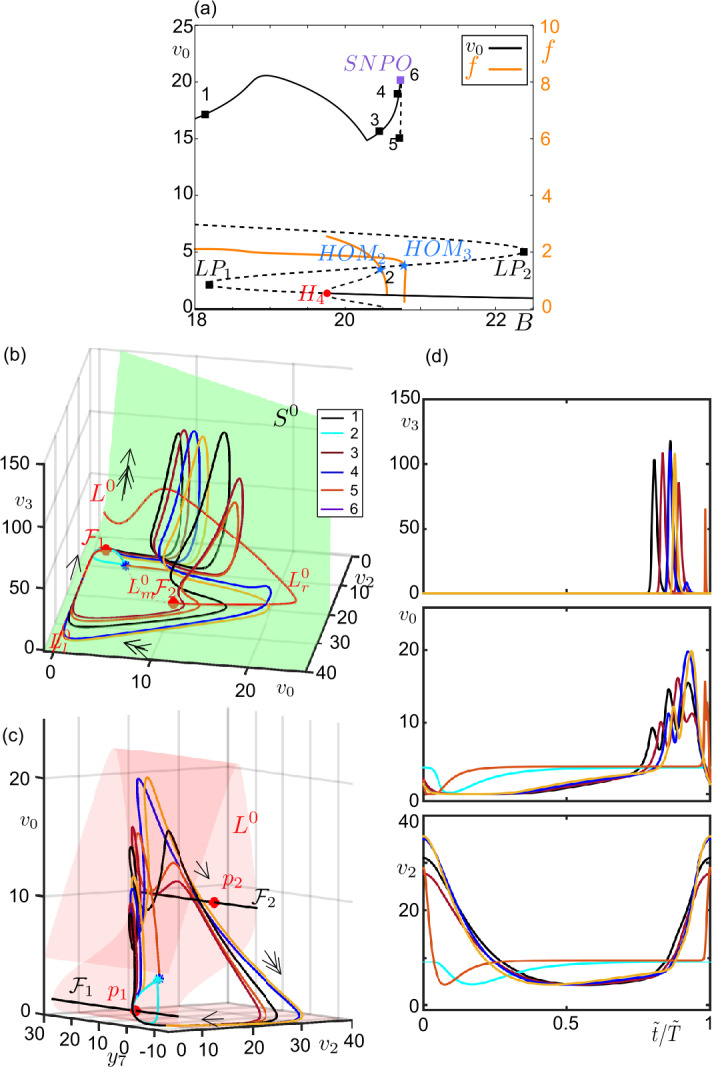
Example canard orbits near the type-I excitable regime. (**a**) Bifurcation diagram for $C_{3} = 50$, $C_{5}=450$ and $B\in [18, 22.5]$ (see Fig. 4c for the whole diagram). Stable and unstable solutions are represented by continuous and dashed curves, respectively. Limit point ($LP_{1,2}$, black squares), Hopf ($H_{4}$, red dot), homoclinic ($HOM_{2,3}$, blue stars) bifurcations and saddle-node bifurcation of periodic orbits (SNOP, orange purple square) are marked on the diagram. Numbered solutions are presented in panels (**b**–**d**). The orange curves trace the frequency of the oscillations. (**b**) Periodic orbits marked in panel (**a**), $L^{0}$ (red curve), fold curves $\mathcal{F}_{1,2}$ (red points) and the critical surface $S^{0}$ (green surface) are projected on the ($v_{0}$, $v_{2}$, $v_{3}$)-space. Arrows indicate the flow direction and its time-scale (single-headed for super-slow, double-headed for slow dynamics. The homoclinic points $HOM_{2}$ and $HOM_{3}$ are marked by cyan and dark blue stars. (**c**) Periodic orbits marked in panel (**a**), $L^{0}$ (red surface), fold curves $\mathcal{F}_{1,2}$ (black curves) and folded singular points $p_{1,2}$ (red dots) are projected on the $(y_{7},v_{2},v_{0})$-space. Arrows indicate the corresponding time-scale. (**d**) Time series of the periodic orbits on panels (**b**, **c**) with respective color codes. Period is normalized to 1 ($\tilde{t}/ \tilde{T} =1$, where *T̃* represents period of a cycle)

Figure 8 zooms in near the excitable region for $C_{3} = 50$ (see Fig. 4c for the whole diagram). For a particular value of *B* for $B < B_{LP1}$, the only attractor is the large amplitude bursting oscillation (the 1st orbit). In $B_{LP1} < B < B_{H4}$ the unstable attractors of the equilibrium points appear. The subcritical Hopf bifurcation at $B = B_{H4}$ initiates a branch of periodic orbits that terminates on the homoclinic point $HOM_{2}$, which bounds the canard explosion near $H_{4}$. For $B_{HOM2}$, a homoclinic canard-without-head orbit (the second orbit) coexists with a large stable bursting orbit of canard type (the third orbit). At $B_{HOM3}$ a homoclinic canard-with-head orbit (the fifth orbit) appears together with an outer large amplitude canard cycle (the fourth orbit). The large amplitude canard cycle grows in amplitude and disappears on a saddle-node of periodic orbits (SNPO) at $B = B_{SNPO}$ (the sixth orbit). We also notice that, as $HOM_{3}$ gets closer to $LP_{2}$ for $C_{3} \approx 54.4$ and $B\approx B_{LP2}$, the canard orbits on the $HOM_{3}$ become of without-head type.

**Figure 9:**
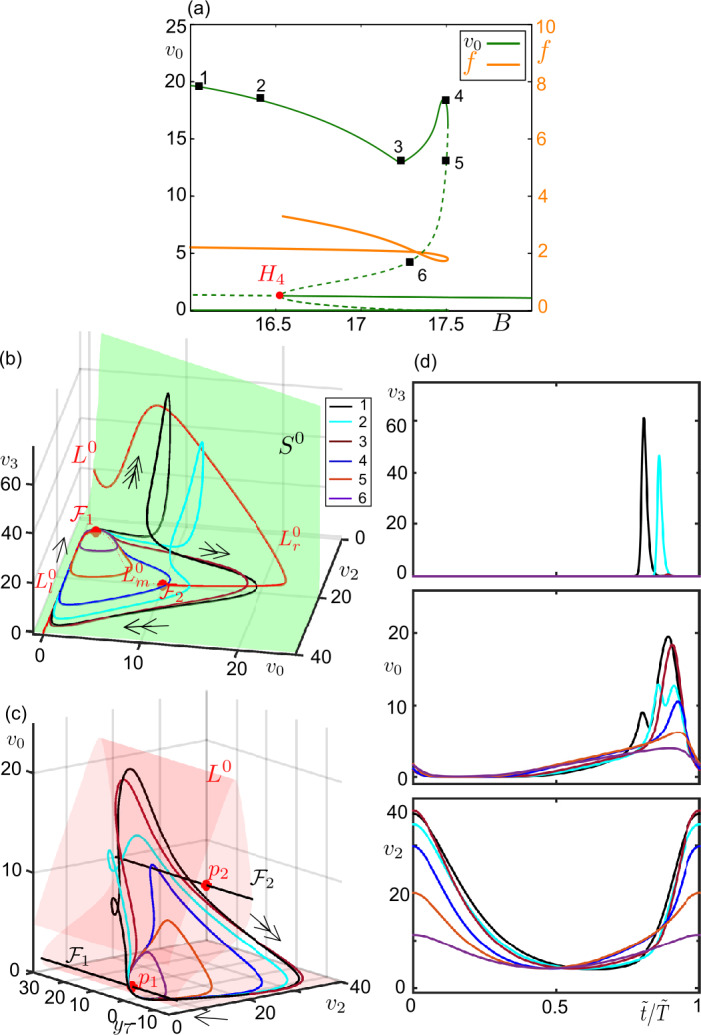
Example canard orbits near the type-II excitable regime. (**a**) Bifurcation diagram for $C_{3} = 80$, $C_{5}=450$ and $B \in [16, 18]$ (see Fig. 5a for the whole diagram). Stable and unstable solutions are represented by continuous and dashed curves, respectively. Hopf bifurcation ($H_{4}$, red dot) is marked on the diagram. Numbered orbits on the lower branch of periodic solutions are presented in panels (**c**–**d**). The orange curve traces the frequency of the oscillations emerging at $H_{4}$. (**b**) Periodic orbits marked in panel (**a**), $L^{0}$ (red curve), fold curves $\mathcal{F}_{1,2}$ (red points) and the critical surface $S^{0}$ (green surface) are projected on the $(v_{0},v_{2},v_{3})$-space. Arrows indicate the flow direction and its time-scale (single-headed for super-slow, double-headed for slow dynamics). (**c**) Periodic orbits marked in panel (**a**), $L^{0}$ (red surface), fold curves $\mathcal{F}_{1,2}$ (black curves) and folded singular points $p_{1,2}$ (red dots) are projected on the $(y_{7},v_{2},v_{0})$-space. Arrows indicate the corresponding time-scale. (**d**) Time series of the periodic orbits on panels (**b**, **c**) are shown with respective color codes. Period is normalized to 1 ($\tilde{t}/ \tilde{T} =1$, where *T̃* represents period of a cycle)

For a parameter set ensuring the type-II excitability ($C_{3} = 80$, for instance), the fast spikes of the bursting oscillations disappear and the final oscillation turns out to be a relaxation type running in slow and super-slow timescales. These relaxation oscillations terminate via a complete canard-explosion near the singular Hopf bifurcation point $H_{4}$. This happens in a similar manner for all $C_{5}$ values under consideration. Figure 9 provides an example for $C_{5}= 450$. As the large amplitude periodic solutions decrease in amplitude, they start to follow $L_{m}^{0}$ and take the shape of canard-with-head solutions (the second and third orbits). The maximal canard of this canard family is the fourth orbit that stays along to the super-slow manifolds as long as possible. After the fourth orbit, we observe canard-without-head orbits (the 5th and the 6th orbits) that shrink to $p_{1}$. The frequency of the oscillations along the canard explosion ranges in $1.8\text{--}3.5\text{ Hz}$. We also notice a region of bistability between large amplitude bursting oscillations and equilibrium points. Once again the canard solutions construct the boundary between them. For a parameter set giving relaxation oscillations in this region (e.g. $C_{5} = \{80, 250, 350\}$ in Fig. 5), the relaxation oscillations shrink $H_{4}$ via a ‘classical’ canard explosion, similar to the one in the 2D van der Pol system, without having any fast component in $v_{3}$.

## Local field potential in critical regimes

**Figure 10:**
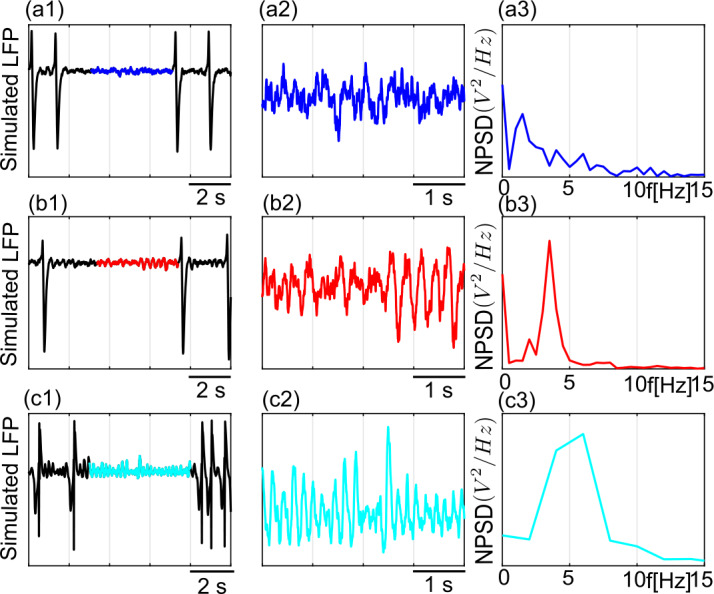
LFP traces of system (1a)–(1h) near critical transitions under stochastic input. (**a1**) Transitions between multiple time-scale oscillations and background regime for a type-I setting at $B=23$, $C_{3} = 35$, $C_{5}=200$. Panel (**a2**) zooms between two large amplitude discharges (blue) and panel (**a3**) shows the normalized power spectral density of the signal. (**b1**) Transitions between multiple time-scale oscillations and background regime with slow oscillations of $\approx 3.5\text{ Hz}$ for a type-II $B=17.8$, $C_{3} = 80$, $C_{5}=200$. Panel (**b2**) zooms between two large amplitude discharges (red) and panel (**b3**) shows the normalized power spectral density of the signal. (**c1**) Transitions between multiple time-scale oscillations and sinusoidal oscillations for $B=4.7$, $C_{3} = 80$, $C_{5}=200$. Panel (**c2**) zooms between two large amplitude discharges (cyan) and panel (**c3**) shows the normalized power spectral density of the signal

**Figure 11:**
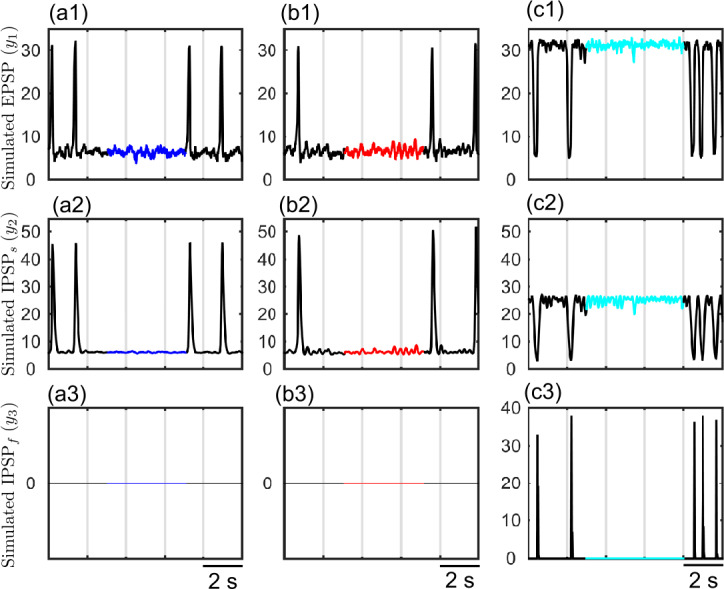
Corresponding PSPs of the LFPs given in Fig. 10. (**a1**)–(**a3**) The EPSP, slow IPSP (IPSP_s_) and fast IPSP (IPSP_f_) for the type-I setting given in Fig. 10a. (**b1**)–(**b3**) The EPSP, slow IPSP (IPSP_s_) and fast IPSP (IPSP_f_) for the type-II setting given in Fig. 10b. (**c1**)–(**c3**) The EPSP, slow IPSP (IPSP_s_) and fast IPSP (IPSP_f_) for the setting given in Fig. 10c

In the previous section we have shown two different regions in parameter space where canard solutions determine boundaries and organize transitions between different dynamical regimes. The narrow-band sinusoidal activity of 4.5–6 Hz emerging near $H_{3}$ and of 1.8–3.5 Hz emerging near $H_{4}$ are connected to large amplitude periodic multiple-timescale solutions through canard orbits. System (1a)–(1h) emits aperiodic large amplitude epileptic discharges under stochastic input ($p(t) = p+ \xi $, with $\xi = \mathcal{N}(0, 2^{2})$) when it is initialized near the critical regions of $H_{3}$ and $H_{4}$ (Fig. 10–11). A parameter setting ensuring the type-I excitability without any canard solutions near $H_{4}$ gives a board band activity between the large amplitude spikes (Fig. 10a1–a3). On the other hand, taking the system to type-II excitability near $H_{4}$ introduces transient small amplitude oscillations of ≈ 3.5 Hz due to the presence of the canard cycles in this region (Fig. 10b1–b3). We observe transitions between large amplitude discharges and harmonic oscillations of ≈ 6 Hz when the system is initialized close to the Hopf bifurcation $H_{3}$ (Fig. 10c1–c3). Simulated PSPs at the level of the pyramidal cell subpopulation are given in Fig. 11.

**Figure 12:**
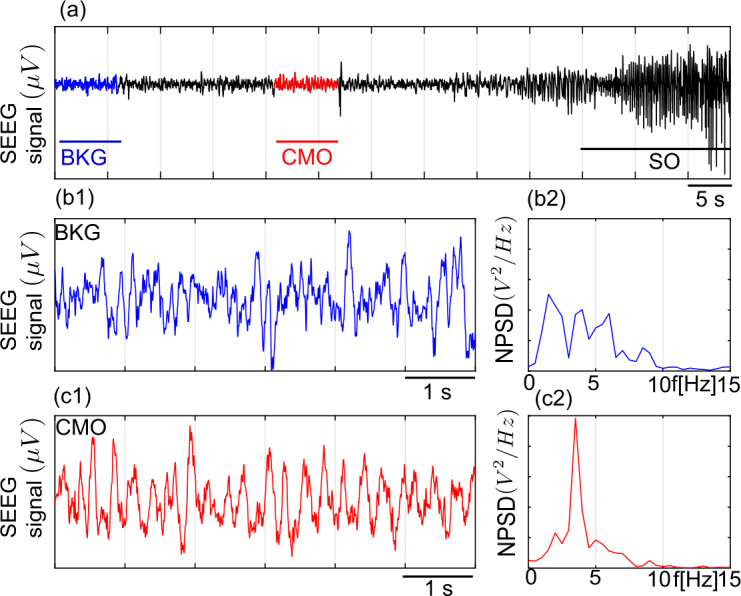
SEEG signals recorded in a patient with epilepsy during the inter-ictal and ictal transition. (**a**) Transition from inter-ictal to ictal period in the first patient. Background activity (BKG) observed further away from seizure (panel b1) has a broad-band frequency distribution (normalized power density spectrum in panel b2). A sporadic spike is preceded by narrow band low amplitude *resembling* canard-mediated oscillations (CMO, marked in red, zoomed in panel (**c1**)) at $\approx 3.5\text{ Hz}$ (normalized power density spectrum in panel (**c2**))

**Figure 13:**
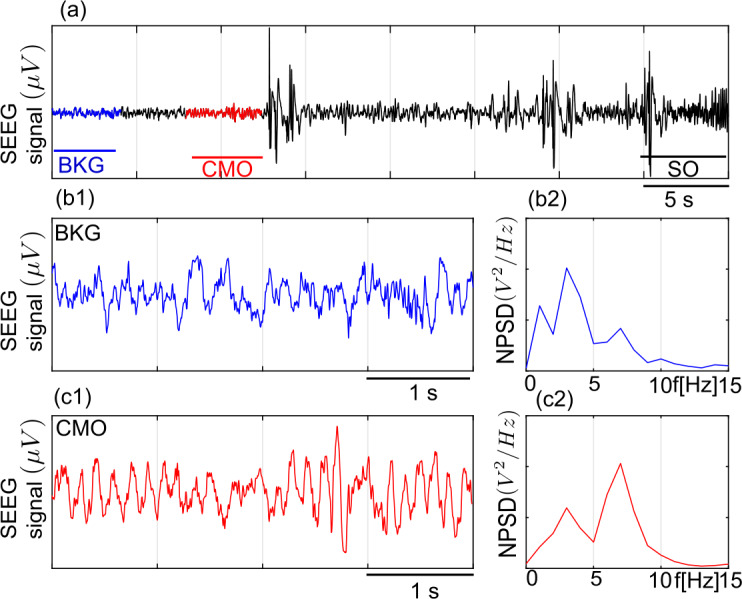
SEEG signals recorded in a patient with epilepsy during the inter-ictal and ictal transition. (**a**) Transition from inter-ictal to ictal period in the second patient. Background activity (BKG) observed further away from seizure (panel **b1**) has a broad-band frequency distribution (normalized power density spectrum in panel **b2**). A sporadic spike is preceded by narrow band low amplitude *resembling* canard-mediated oscillations (CMO, marked in red, zoomed in panel (**c1**)) at $\approx 7\text{ Hz}$ (normalized power density spectrum in panel (**c2**))

Figures 12 and 13 show LFPs recorded by the SEEG electrodes in two different patients with drug-resistant focal epilepsy during presurgical evaluation (see Table 2 for the details). Multiple-contact depth electrodes were implanted according to the SEEG technique as a standard clinical procedure in the care of patients who consented the possible use of data for research purpose. The positioning of the electrodes is determined in each patient from hypotheses about the localization of the epileptogenic areas. Implantation accuracy peri-operatively is verified by an X-ray CT scan. A post-operative CT scan without contrast product is then used to verify the precise 3D location of each electrode contact. After SEEG exploration, intracerebral electrodes are removed. An MRI is performed on which the trajectory of each electrode remains visible. Finally, a CT-scan/MRI data fusion is performed to anatomically locate each contact along each electrode trajectory. The patient had electrodes implanted in the temporal region. For this study, signals were selected as they exhibited clear transitions in electrophysiological patterns. In particular, we selected pre-ictal events followed by a fast discharge typical of the seizure onset, which is one of the markers of the imbalanced relation of excitation and inhibition [16, 71] that involves excitability variations.

**Table 2:** Summary of patients’ features

Feature	Patient 1 (Fig. 12)	Patient 2 (Fig. 13)
Age at SEEG	16 y	14 y
Gender	Female	Female
MRI	Right occipito-temporal focal cortical dysplasia	Left hippocampal sclerosis
Syndrome	Temporal lobe epilepsy (temporal plus)	Mesial temporal lobe epilepsy
Recorded Cerebral Region	Medial part of the middle temporal gyrus (electrode contact: B4)	Internal temporal pole (electrode contact: Pt’1)
Surgical outcome (Engel Class)	II (cortectomy)	IA (anterior temporal lobectomy)

In Fig. 12, a narrow band activity of theta-band of 3.5 Hz is followed by a large amplitude epileptic discharge between two sporadic discharges as we advance towards sustained pre-ictal discharges. Such narrow band activity may be a signature of canard-mediated regions where slowly varying system’s parameters and/or remote interactions lead to transitions between small-amplitude-low-frequency oscillations and large amplitude discharges. In Fig. 13 a narrow band activity of about 7 Hz is followed by a large amplitude epileptogenic discharge. We also notice that the form of the epileptic discharges in Fig. 12 and Fig. 13 are different, which may indicate that the systems would have different characteristics. Interestingly, we have identified parameter regions for the corresponding frequency bands in the model (see simulated LFPs and PSPs in Fig. 10 and Fig. 11, respectively). Hence, we think that the properties of transient narrow band oscillations may be related to the excitability properties and level of synaptic projections (scaled by the coupling coefficients in the model) of the epileptogenic zone.

## Conclusion

In this article, we extended the multiple time-scale analyses previously initiated in [48]. Here, we both investigated canard transitions present in a neurophysiologically-relevant NMM and analyzed their consequences in terms of subsequent signatures in LFPs. In this three-time-scale model, the canard transitions occur in the 6-dimensional two-time-scale reduced system of slow and super-slow variables. They are associated with degenerate FSN II singularities and singular Hopf bifurcations. They organize initiation of relaxation/bursting oscillations from harmonic oscillations of 4.5–6 Hz or from equilibrium points, and determine the boundaries between them. We showed that the system switches between type-I and type-II excitability near the transitions between the equilibrium points and relaxation/bursting oscillations. We further noticed that the canard regimes of type-II excitability (and partially of type-I) yield low-frequency (near 3.5 Hz) oscillations in the LFP under stochastic input.

These model predictions motivated a close analysis of SEEG recordings performed in epileptic patients. In this paper, results illustrative of both signatures are reported only in two patients. Interestingly, in brain structures clearly involved in the transition from interictal to ictal activity, we observed a narrow band activity between sporadic discharges before the seizure initiation, which strongly differed from the preceding background activity. Although the parameter set used in this paper was not aimed for modeling these recordings specifically, it is striking to see such a matching between the mathematical analysis and the actual recordings.

It has been evidenced that impaired excitation–inhibition balance shapes the activity of neural networks and, therefore, causes the emergence of “pathological” electrophysiological patterns such as pre-ictal spikes and seizures in the context of epilepsy (see for a review [72]). Indeed, epileptogenic brain regions are typical example of such excitation–inhibition imbalanced networks [73]. We showed that the level of EPSP on the subpopulation of SOM+ interneurons determines the type of the excitability. In particular, the system is type-I excitable if the average number of synaptic contacts from the excitatory pyramidal cells to the GABAergic SOM+ interneurons is low, and type-II excitable if the average number of synaptic contacts from the excitatory pyramidal cells to the GABAergic SOM+ interneurons is high. It is then the decreasing GABAergic inhibition (modeled by decreasing inhibitory drive by the subpopulation of the SOM+ interneurons) that is responsible for transitions from background to epileptiform discharges. Interestingly, such model parameter variations are plausible and linked to the failure of inhibitory barrages observed in epileptic tissues [74] and generation of slow waves preceding the fast activity [75, 76]. Properties of emerging epileptic discharges (e.g. their shape and frequencies), and possible “silent” phases in between are strongly connected to the type of the excitability. In the context of epilepsy, transitory regimes between the background activity and epileptic discharges are crucial for understanding the underlying mechanisms [11, 77]. Epileptic biomarkers during such regimes, such as high-frequency oscillations [78], shape features of epileptic spikes [79] or maybe frequency-specific oscillations reported here, are essential for identification of epileptogenic networks and for further development of therapeutic procedures. Verification of the presence of such oscillations across different patients and accurate modeling of the inter-ictal activity are needed, of course, for suggesting them as biomarkers. This is the topic of future investigations.

As epilepsy can be considered as a dynamic disease [73, 80, 81], mathematical models of different cellular levels inherit multiple time-scale thinking [82–84]. We note a few studies on the slow–fast transitions in NMMs. Desroches et al. extended NMMs [85] by considering the synaptic gain of SOM+ interneurons as a slowly changing variable. They showed that this configuration introduced regions of torus canards. Jafarian et al. [86] proposed a NMM which incorporates slow variations in ionic currents leading to spontaneous paroxysmal activity. Hebbink et al. [87] investigated response of the NMM of Wendling et al. [16] to slowly varying inputs under which the systems yields bursting oscillations. Weigenand et al. remarked the role of canard solutions in fast transitions in sleep wake patterns of K-complexes in a NMM of sleep-wake patterns [43]. Our paper shows that canard-mediated solutions are naturally present in the NMM of Wendling et al. [16]. Importantly, as this model implements two main sub-types of interneurons (dendrite- and soma-projecting), it is generic and can be considered for studying the dynamics of other regions than hippocampus, such as neocortical areas, and in different contexts, such as consciousness [47] and Alzheimer’s disease [20]. Furthermore, canard regimes reported in this study are governed by the interactions between the pyramidal cell and SOM+ interneuron subpopulations that follow a two-time-scale structure. It would be natural to observe canard-mediated transitions in another generally used NMM of Jansen and Rit [13] for modeling the brain activity. Hence, the canard-mediated fine structures we have demonstrated here could be relevant for a number of situations and lead to markers of subsequent critical transitions. The reported degenerate FSN II singularity leading to canard trajectories is due to the general structure of NMMs, which are defined via second-order differential equations. The dynamics associated with the degenerate FSN II singularity merits further investigations and will be considered as a future work. Finally, organisation of homoclinic canard orbits, possible codimension-two bifurcations and interactions with the fold points will be studied in forthcoming works.

## Data Availability

The codes used for numerical analysis are available from the GitHub database (https://github.com/elifkoksal/NMM_BurstingDynamics).

## Abbreviations

NMM: Neural Mass Model
EEG: Electroencephalography
LFP: Local Field Potential
GSPT: Geometric Singular Perturbation Theory
SOM+: Somatostatin positive
PV+: Parvanium positive
CA1: Cornu Ammonis 1
PSP: Post-Synaptic Potential
EPSP: Excitatory Post-Synaptic Potential
IPSP: Inhibitory Post-Synaptic Potential
SEEG: Stereoelectroencephalography
FSN II: Folded Saddle-Node type II
DSRS: Desingularized Slow Reduced System
LP: Limit Point
H: Hopf
HOM: Homoclinic
CP: Cusp Point
BT: Bogdanov–Takens
GH: Generalized Hopf
SNIC: Saddle-Node of Invariant Cycle
SNPO: Saddle-Node of Periodic Orbits
